# Synthesis of protein conjugates adsorbed on cationic liposomes surface

**DOI:** 10.1016/j.mex.2020.100942

**Published:** 2020-05-28

**Authors:** Despo Chatzikleanthous, Robert Cunliffe, Filippo Carboni, Maria Rosaria Romano, Derek T. O'Hagan, Craig W. Roberts, Yvonne Perrie, Roberto Adamo

**Affiliations:** a Strathclyde Institute of Pharmacy and Biomedical Sciences, University of Strathclyde, 161 Cathedral St, G4 0RE Glasgow, UK; b GSK, Via Fiorentina 1, 53100 Siena, Italy; c GSK, 14200 Shady Grove Rd, Rockville, MD, United States

**Keywords:** TLR9 agonist, Conjugation, Cationic liposomes, Surface adsorption, Nanoparticles, Vaccines, Group B Streptococcus, Neisseria meningitidis

## Abstract

The well-known Toll like receptor 9 (TLR9) agonist CpG ODN has shown promising results as vaccine adjuvant in preclinical and clinical studies, however its *in vivo* stability and potential systemic toxicity remain a concern. In an effort to overcome these issues, different strategies have been explored including conjugation of CpG ODN with proteins or encapsulation/adsorption of CpG ODN into/onto liposomes. Although these methods have resulted in enhanced immunopotency compared to co-administration of free CpG ODN and antigen, we believe that this effect could be further improved. Here, we designed a novel delivery system of CpG ODN based on its conjugation to serve as anchor for liposomes. Thiol-maleimide chemistry was utilised to covalently ligate model protein with the CpG ODN TLR9 agonist. Due to its negative charge, the protein conjugate readily electrostatically bound cationic liposomes composed of 1,2-distearoyl-sn-glycero-3-phosphocholine (DSPC), cholesterol and dimethyldioctadecylammonium bromide (DDA) in a very high degree. The novel cationic liposomes-protein conjugate complex shared similar vesicle characteristics (size and charge) compared to free liposomes. The conjugation of CpG ODN to protein in conjunction with adsorption on cationic liposomes, could promote co-delivery leading to the induction of immune response at low antigen and CpG ODN doses.

• The CpG ODN Toll-like receptor (TLR) 9 agonist was conjugated to protein antigens via thiol-maleimide chemistry.
• Due to their negative charge, protein conjugates readily electrostatically bound cationic liposomes composed of 1,2-distearoyl-sn-glycero-3-phosphocholine (DSPC), cholesterol and dimethyldioctadecylammonium bromide (DDA) resulting to the design of novel cationic liposomes-protein conjugate complexes.
• The method is suited for the liposomal delivery of a variety of adjuvant-protein conjugates.

## Graphical abstract

**Figure:**
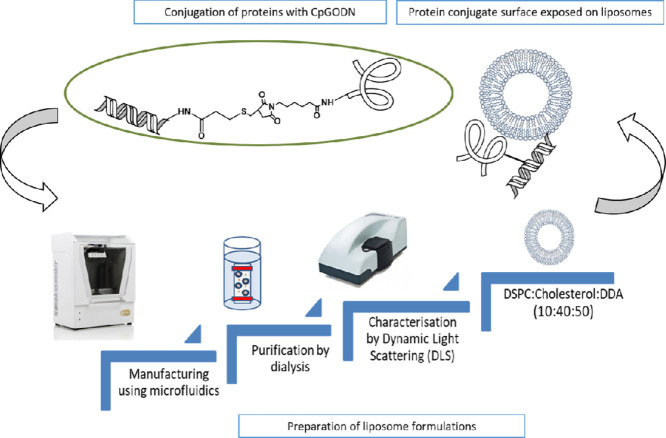

Specifications Table

**Table:** 

Subject Area:	Pharmacology, Toxicology and Pharmaceutical Science
More specific subject area:	Vaccine delivery systems
Method name:	Adsorption of CpG-protein conjugates on the surface of cationic liposomes
Name and reference of original method:	Conjugation of CpG ODN on protein antigens [1,2]
	Manufacturing of liposomes with microfluidics [3,4]
	Adsorption of proteins and adjuvants on liposomes surface [5]
Resource availability:	

## Method details

### Overview

CpG oligodeoxynucleotides (CpG ODN) are short single-stranded synthetic DNA molecules that include of a cytosine triphosphate deoxynucleotide and a guanine triphosphate deoxynucleotide. They mimic microbial DNA that often contains these motifs and their ability to enhance immune responses is well documented [6,7] Responsiveness to CpG motifs is mediated through TLR9, a receptor localised to and signalling from the endosomal compartment of antigen presenting cells (APCs), such as dendritic cells (DCs) and macrophages. TLR9 binding of CpG-containing DNA results in the induction of rapid innate immune responses to prevent or limit early infection, but crucially also directs the quality of the specific adaptive immune response to facilitate pathogen clearance and, finally, memory responses for long-lived protection. Supported by the induction of immunostimulatory T helper Th1-biasing cytokines and chemokines including interleukin IL-12, tumour necrosis factor TNF-α and interferon IFN α/β and γ, CpGs directly (i.e., APCs) or indirectly (i.e. natural killer cells and T lymphocytes) activate a variety of immune cells, ultimately resulting in enhanced immune function [8].

TLR9 agonist CpG ODN has shown promising results as a vaccine adjuvant in preclinical and clinical studies [9]. Despite this success, the use of CpG ODN is associated with several obstacles including poor in vivo stability mainly due to their digestion by endonucleases, unfavourable pharmacokinetic and biodistribution profiles and poor cellular uptake characteristics [6]. In addition, there are safety concerns regarding undesirable side effects observed depending on the administered dose [10]. These side effects include liver toxicity, enlargement of the lymph nodes [11], extramedullary hematopoiesis [12], systemic inflammation [9,^13^,14] and renal damage [15]. Additionally, autoimmune responses have been observed in cancer patients [16]. Reduction in such effects could be achieved by lowering the dose of administered compound.

In an effort to circumvent these issues, alternative *in vivo* delivery systems of CpG ODN, including conjugation strategies and nanoparticulate formulations, have been suggested. Conjugation of CpG motifs with protein antigens creates a more potent immunogen compared to physical mixture of antigen and CpG [17]. Co-localisation, improved antigen uptake and presentation, and thus enhanced immune responses are some of the benefits of such protein conjugates. Specifically, whilst protein-CpG mixtures have the limitation of inconsistent co-localisation, protein-CpG conjugates ensure efficient internalisation of antigen and adjuvant by the same DCs through endocytosis and activation of the intracellular TLR9, allowing the use of lower doses of adjuvant compared to the unconjugated form [18], [19], [20]. As an alternative to conjugation, liposomal delivery of CpG ODN has been demonstrated to offer important advantages including protection from DNase degradation, extension of retention time inside the body, improved cellular uptake, delivery to target tissues and slow release over a long period of time [21]. Various types of liposomal CpG ODN have been developed to achieve immunostimulation, and encapsulation or co-administration of CpG motifs into/with liposomes have been shown to dramatically enhance the potency of immunogens compared to free CpG ODN [22,^23^,^24^,25]. Special focus has been given on the use of cationic liposomes as their positive charge favours formation of the depot effect at the injection site [26] thus improving the antigen presentation to APCs followed by a sustained release to the draining lymph nodes [27].

In this context, we explored the potential of protein-CpG ODN conjugate anchored to liposome nanoparticles by adsorption to enhance immunogenicity. It was anticipated that the covalent linkage of the TLR9 agonist CpG ODN to a protein antigen multivalently presented on the surface of cationic liposomes could promote accumulation of protein and adjuvant within the body, facilitate their delivery and further increase vaccine efficiency compared to protein conjugation alone or liposome delivery.

## Materials

CpG ODN 1826 (5‘-[AmC6]TCCATGACGTTCCTGACGTT), N-ε-malemidocaproyl-oxysuccinimide ester (EMCS), succinimidyl 3-(2-pyridyldithio)propionate (SPDP), Tris(2-carboxyethyl)phosphine hydrochloride solution (TCEP), sinapinic acid and OVA were purchased from Sigma-Aldrich (Poole, Dorset, UK). Cholesterol, 1,2-distearoyl-sn-glycero-3-phosphocholine (DSPC), Dimethyldioctadecylammonium (DDA) were purchased from Avanti Polar Lipids (Alabaster, AL, USA). GBS67, NadA, CRM197 were supplied by GSK (Siena, Italy).

### Chemical synthesis

CpG ODN was conjugated to three different proteins: Cross-reactive material 197 (CRM197), Neisseria adhesin A (NadA), Group B Streptococcus 67 (GBS67). CRM197 (MW 58 kDa, pI=5.85) is an enzymatically inactive and nontoxic form of diphtheria toxin found to be an ideal carrier for conjugate vaccines against encapsulated bacteria [28], [29], [30]. NadA (MW 25 kDa, pI=4.4) is a surface exposed trimeric protein presented in approximately 50% of pathogenic meningococcal isolates and is associated mostly with strains that belong to three of the four hypervirulent serogroup B lineages. NadA is the most well characterised and known antigen between the ones included in Bexsero vaccine and for this reason has been selected as model antigen for this study [31]. GBS67(MW 98 kDa, pI=6.46) is an ancillary highly conserved protein of pilus 2a [32], [33], [34]. Pilus proteins have been identified through reverse vaccinology as promising vaccine candidates [35]. The conjugation was achieved through the well-known thio-maleimide click reaction [1,^2^,^19^,36].

### Preparation of protein-EMCS

For the incorporation onto the protein of maleimides moieties, 1.52 mg of EMCS were dissolved in 50 µL of DMSO, and 11 µL of the prepared mixture (6 eq-v) was added to a solution of 6 mg of protein (CRM197: 130 µL of 47.4 mg/mL stock solution, NadA: 860 µL of 7 mg/mL stock solution, GBS67: 330 µL of 18.4 mg/mL stock solution) in 100 mM sodium phosphate (NaPi), 1 mM EDTA pH 8.1 buffer solution (Final volume 1 mL). Reaction was incubated for 3 h at RT. After 3 h, reaction mixture was purified using 30 kDa Viva spin filter units 0.5 mL (x5 dialysis cycles) dialysing against 50 mM NaPi, 1 mM EDTA pH 7.5. Protein content was determined by BCA colorimetric assay. The linker/protein molar ratio was determined by MALDI-TOF mass spectrometry analysis run in an UltraFlex III MALDI-TOF/TOF instrument (Bruker Daltonics, Bremen, Germany) in linear mode and with positive ion detection. The sample for analysis was prepared by mixing 2.5 µL of product and 2.5 µL of sinapinic acid matrix. 2.5 µL of mixture was deposited on a sample plate, dried at RT for 10 min, and subjected to the spectrometer.

### Preparation of CpG ODN-SH

An amount of 20 mg (3.21 µmol) of CpG ODN 1826 (5‘-[AmC6] TCCATGACGTTCCTGACGTT, MW 6238) was reacted with 10 eq-v (10 mg, 32.1 µmol) of SPDP linker in 1:9 v/v 100 mM NaPi pH 7.2: DMSO (1 mL). The reaction mixture was incubated for 3 h at RT under continuous mixing and was purified by size exclusion chromatography on a G25 Sephadex column eluting with H_2_O. Fractions contained the CpG ODN-SH were combined and concentrated by Genevac evaporator (Genevac, Ipswich, Suffolk, UK). ^1^H NMR was performed in order to assess the incorporation of the linker. To release the free thiol groups, CpG ODN-SH was treated with 3 eq-v of 0.0005 M TCEP solution for 3 h at RT in the dark. The reaction mixture was purified by size exclusion chromatography using G25 Sephadex column and H_2_O as eluent. The amount of CpG ODN-SPDP recovered was quantified by measuring UV absorbance at 260 nm.

### Conjugation of CpG ODN-SH to protein-EMCS

Protein conjugate was prepared by incubating protein-EMCS with CpG ODN-SPDP (1:10 eq-v protein: CpG ODN) in 50 mM NaPi, 1 mM EDTA pH 7.5 (Final volume 500 µL). Table 1 presents the volumes used for each protein conjugation. The reaction was incubated overnight at RT under continuous mixing. Protein conjugate was purified using 30 kDa Vivaspin filter unit 0.5 mL (x 40 dialysis cycles) and recovered in PBS (1x) buffer. The protein and CpG ODN content were determined by BCA colorimetric assay and UV absorbance (260 nm), respectively. Finally, the extent of protein conjugation to CpG ODN was evaluated by SDS-PAGE electrophoresis and SEC—HPLC. The standard SEC protocol was carried out using TSKgel G4000SW column (300 × 7.8 mm, 500 Ǻ, 5 µm particle size) from Tosoh Bioscience (Tokyo, Japan). Running conditions were flow rate 0.5 mL/min, run time 45 min, 100 mM NaPi, 100 mM Na_2_SO_4_, ACN 5%, pH 7.1 as running buffer and injection volume 50 µL. All samples were injected in a protein concentration of 0.5 mg/mL for protein and protein-EMCS and protein-EMCS-CpG ODN and 0.5 mg/mL for free CpG.

**Table 1:** Volumes and quantities used for conjugations.

Conjugate	Protein-EMCS used	CpG ODN-SPDP used
CRM197-CpG ODN	4.8 mg (100 µL of 48.2 mg/mL stock solution)	5,23 mg (dissolved in 50 mM NaPi, 1 mM EDTA pH 7.5)
NadA-CpG ODN	3 mg (280 µL of 10.7 mg/mL stock solution)	2,54 mg (dissolved in 50 mM NaPi, 1 mM EDTA pH 7.5)
GBS67-CpG ODN	4 mg (350 µL of 11.4 mg/mL stock solution)	2,7 mg (dissolved in 50 mM NaPi, 1 mM EDTA pH 7.5)

### Preparation of liposomes bearing protein-CpG ODN conjugate

The preparation of DSPC: Cholesterol: DDA cationic liposomes was achieved via microfluidics (Nanoassemblr, Precision NanoSystems Inc., Vancouver, Canada) processes based on previously developed methods [3,^4^,37]. Briefly, DSPC: Cholesterol: DDA lipid stock mixture was prepared in ethanol at 10:40:50 molar ratio (2.88 mg/mL DSPC, 5.63 mg/mL cholesterol, 11.49 mg/mL DDA). Then, the lipids and an aqueous phase (10 mM TRIS buffer pH 7.4) were injected simultaneously in the micromixer. The volumes of lipid (organic) and aqueous phase injected depends on the manufacturing conditions have been selected. Herein, all formulations were prepared at 20 mg/mL initial lipid concentration, 1:1 v/v aqueous: organic flow rate ratio (FRR) and 12 mL/min total flow rate (TFR). All newly formed liposomes (1 mL) were then subjected to buffer exchange via dialysis against 10 mM TRIS pH 7.4 for 1 h under magnetic stirring to ensure removal of residual solvent.

To investigate the adsorption of protein-CpG ODN conjugate onto the surface of liposomes, protein-CpG ODN was mixed with DSPC: Cholesterol: DDA liposomes to a similar manner as reported before [5]. Briefly, liposomes were incubated with protein-CpG ODN (1:20 w/w protein: liposomes) in 10 mM TRIS pH 7.4. To serve as controls, liposomes adsorbed free protein or free protein and CpG ODN were also prepared. Samples were left to equilibrate for 30 min at RT. Dialysis using Biotech CE tubing (300 kDa MWCO) was carried out overnight at 4 °C with two buffer changes, for removal of unbound protein. BCA assay and UV (260 nm) were used for quantification of protein and CpG ODN, respectively. The amount of protein adsorbed on liposomes surface was calculated by subtracting the amount of protein remaining in solution from the amount of protein initially added to the liposome dispersion. OVA (MW 45 kDa, pI=4.5) served as positive control as its behaviour in the presence of various liposome formulations is well established.

### Liposome characterisation

The size distribution (mean diameter and polydispersity index (PDI)) and the zeta potential of the liposomes were measured by dynamic light scattering using photon correlation spectroscopy on a Zetasizer Nano-ZS (Malvern Instruments Ltd., UK). Measurements were made at 25 °C with liposomes being diluted in 1/10 v/v using their aqueous phase (1:300 v/v 10 mM TRIS pH 7.4). Sizes quoted are the z-average mean for the liposomal hydrodynamic diameter (nm).

## Method validation

### Protein-TLR9 agonist conjugate assembly

**Fig. 1:**
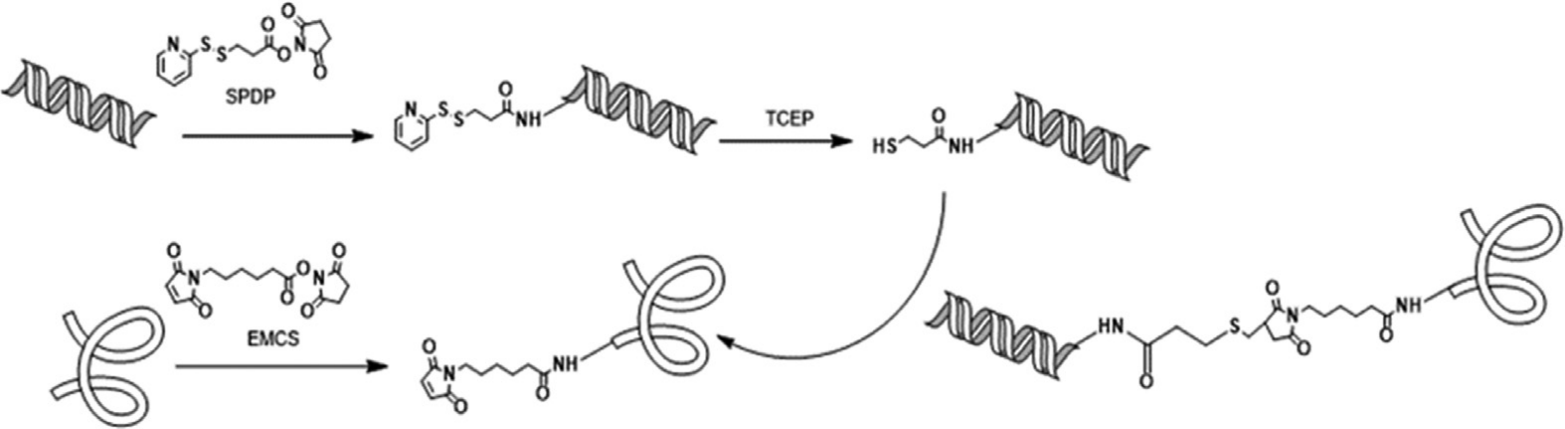
Reaction scheme for conjugation of CpG ODN on proteins.

CpG ODN-protein synthesis was achieved in a similar manner to that reported for other adjuvant-protein conjugates [1,^2^,^19^,38]. Maleimide groups were inserted onto protein by reaction of the protein with commercial EMCS linker. An incorporation of 4–6 maleimides were found by MALDI-TOF analysis of the modified proteins (Fig. S1 Supporting material) [1,39]. Thiol groups were introduced onto CpG ODN 1826 by reaction of the primary amine at 5′ position of the adjuvant molecule with the active ester of SPDP linker. ^1^H NMR analysis confirmed the successful modification of CpG ODN (Fig. S2 Supporting material). After removal of the thio-pyridine protection with TCEP, CpG ODN bearing the sulfhydryl groups was incubated with protein-EMCS to give addition to the maleimides exposed onto the protein surface (Fig. 1).

**Fig. 2:**
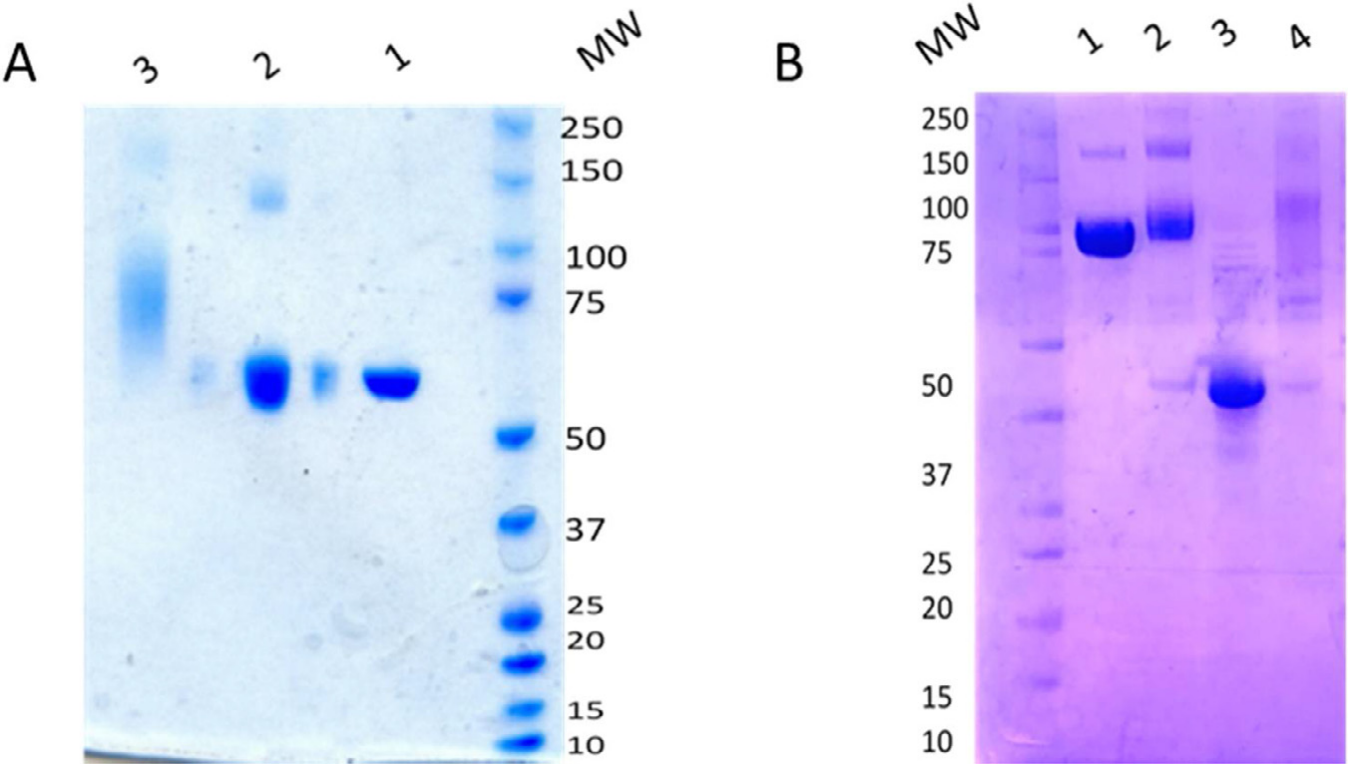
SDS-PAGE for confirmation of protein-CpG ODN conjugation. A) Bands: 1. CRM197, 2. CRM197-EMCS, 3. CRM197-EMCS-SPDP-CpG ODN B) Bands: 1. GBS67, 2. GBS67-EMCS-SPDP-CpG ODN, 3. NadA, 4. NadA-EMCS-SPDP-CpG ODN.

**Fig. 3:**
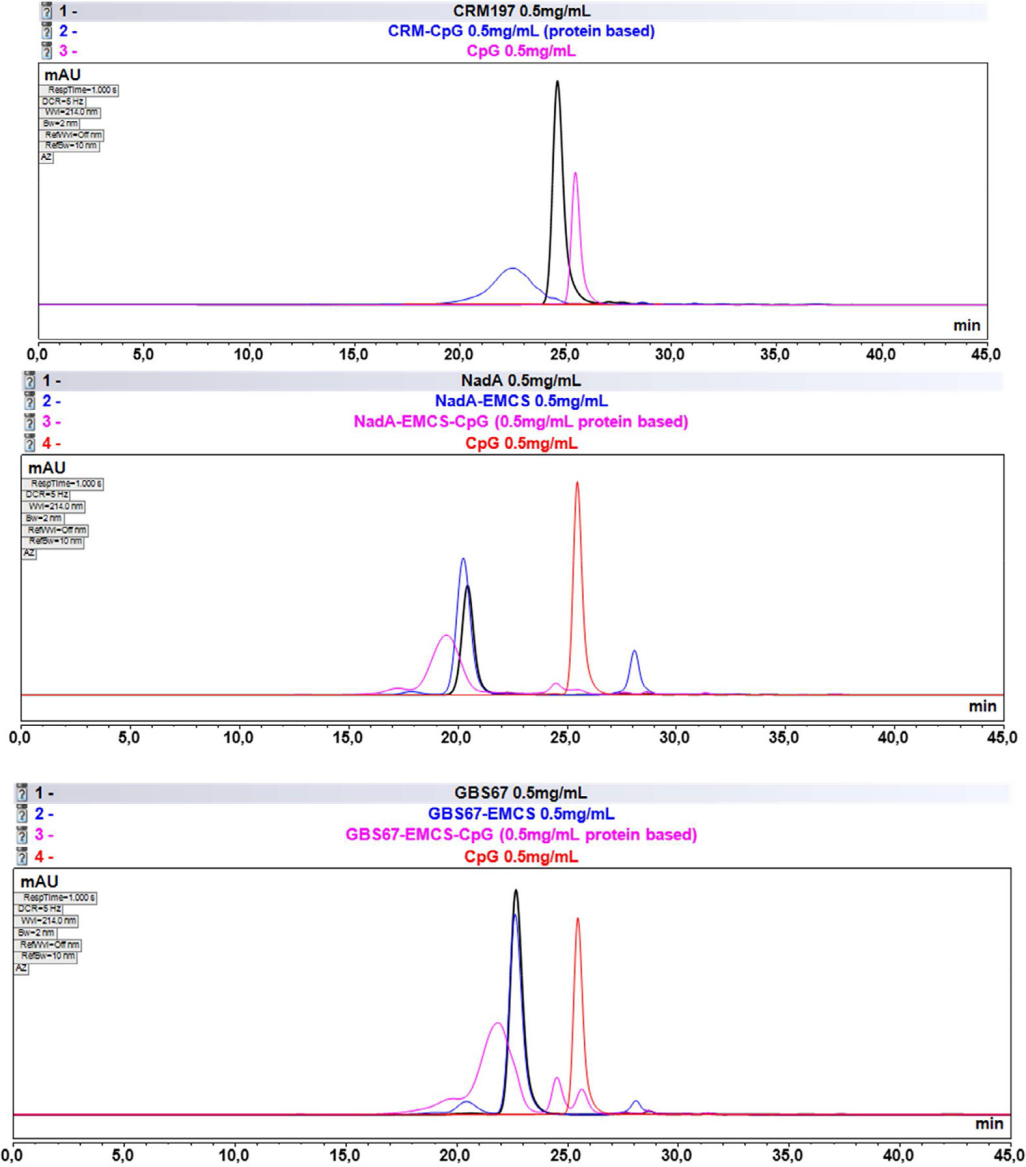
SEC—HPLC for confirmation of protein-CpG ODN conjugation. Experiments performed using a TSKgel G4000SW column (300 × 7.8 mm, 500 Ǻ, 5 µm particle size) from Tosoh Bioscience (Tokyo, Japan). Running conditions were flow rate 0.5 mL/min, run time 45 min, 100 mM NaPi, 100 mM Na_2_SO_4_, ACN 5%, pH 7.1 as running buffer and injection volume 50 µL. All samples were injected in a protein concentration of 0.5 mg/mL for protein and protein-EMCS and protein-EMCS-CpG ODN and 0.5 mg/mL for free CpG.

SDS-PAGE electrophoresis and SEC—HPLC clearly showed conjugation of the CpG ODN to the modified protein (Figs. 2, 3). An average ratio of 4–6 CpG ODN chains was incorporated for each protein molecule in the final product which is in agreement with what has been previously published for the preparation of CpG ODN conjugates using other proteins [19,36]. The characteristics of the protein conjugates are summarised in Table 2.

**Table 2:** Introduction of CpG ODN chains on proteins.

Structure	CpG ODN: protein stoichiometry (mol/mol)	MW protein-CpG ODN conjugate	CpG ODN: protein in conjugate (mol/mol)	a Conjugation efficiency (%)
CRM197	10:1	100,000	6:1	60%
NadA	10:1	100,000	4:1	40%
GBS67	10:1	120,000	4:1	40%

a Amount of conjugated CpG ODN vs amount of CpG ODN used for conjugation.

### Association of protein with liposomes

**Fig. 4:**
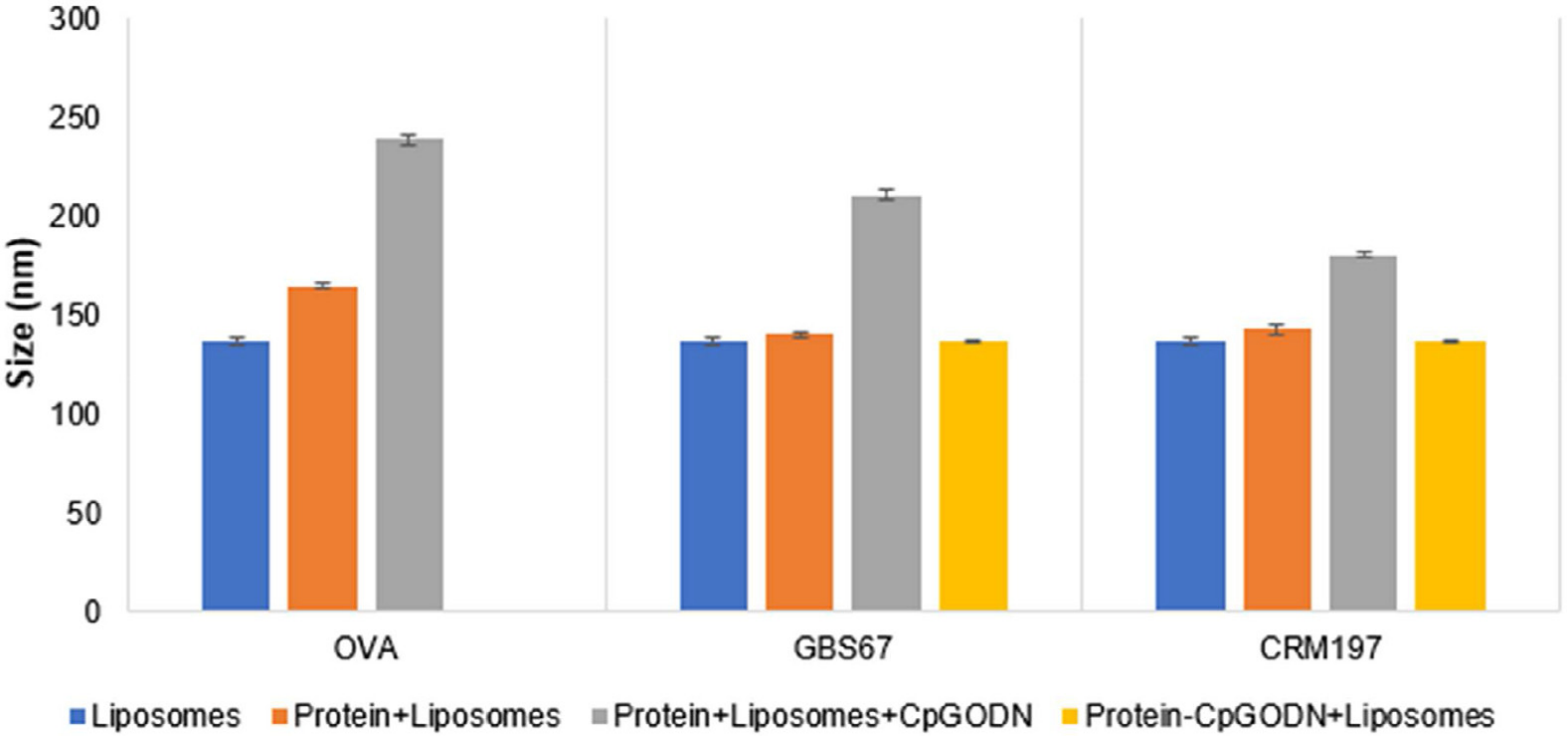
The effect of CpG ODN loading on size of liposomes. DSPC:Cholesterol:DDA (10:40:50% molar ratio) liposome were manufactured using microfluidics at 1:1 FRR, 12 mL/min TFR and purified using dialysis. Liposomes were mixed with free protein, protein+CpG ODN mixture or protein-CpG ODN conjugate and purified by dialysis. The final liposome (5 mg/mL), protein (0.25 mg/mL) and CpG ODN (0.038 mg/mL) concentrations in all the samples were the same. Liposomes were characterised in terms of size and PDI by DLS. Results represent mean ± SD, n = 3 independent batches.

**Fig. 5:**
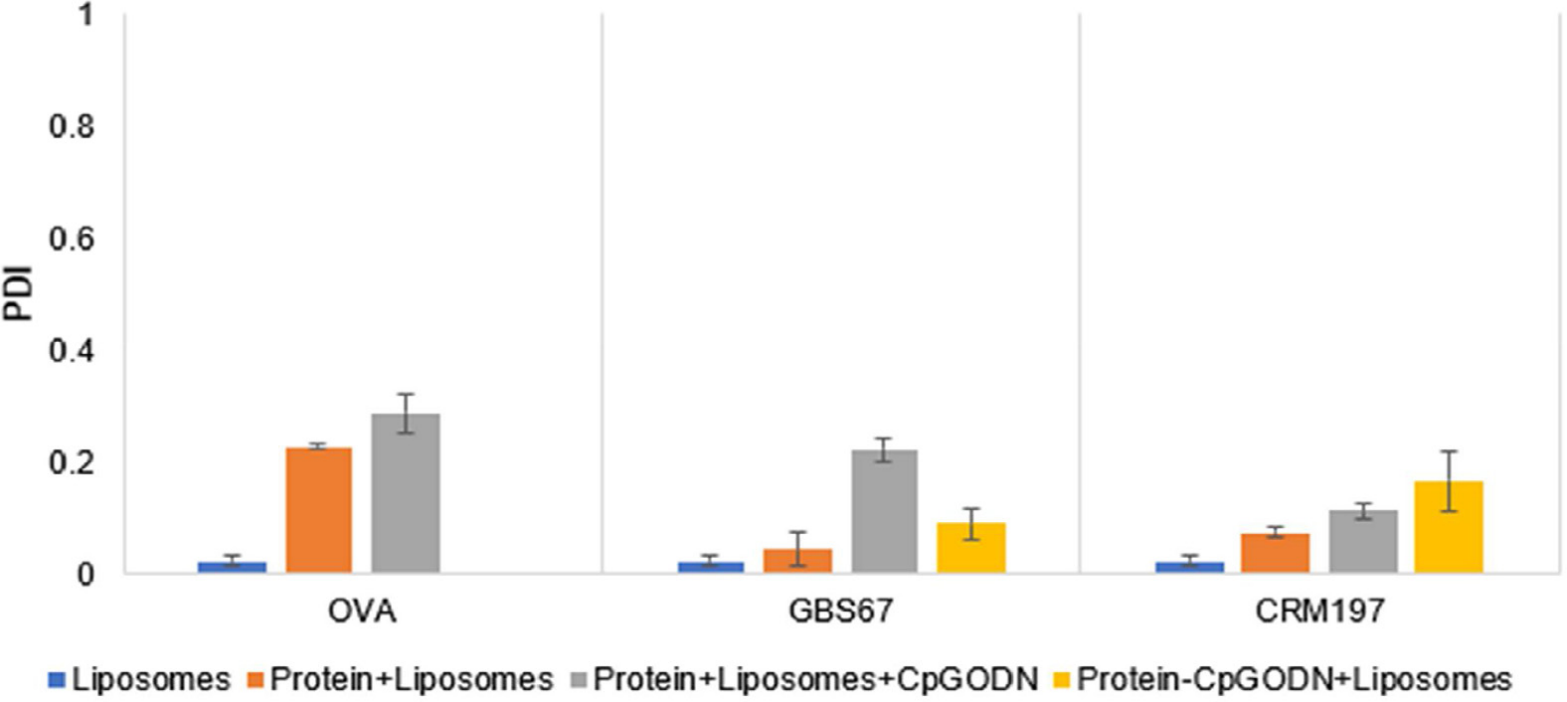
The effect of CpG ODN loading on PDI of liposomes. DSPC:Cholesterol:DDA (10:40:50% molar ratio) liposome were manufactured using microfluidics at 1:1 FRR, 12 mL/min TFR and purified using dialysis. Liposomes were mixed with free protein, protein+CpG ODN mixture or protein-CpG ODN conjugate and purified by dialysis. The final liposome (5 mg/mL), protein (0.25 mg/mL) and CpG ODN (0.038 mg/mL) concentrations in all the samples were the same. Liposomes were characterised in terms of size and PDI by DLS. PDI is a measure of the monodispercity of sizes of particles in the mixture. Results represent mean ± SD, n = 3 independent batches.

**Fig. 6:**
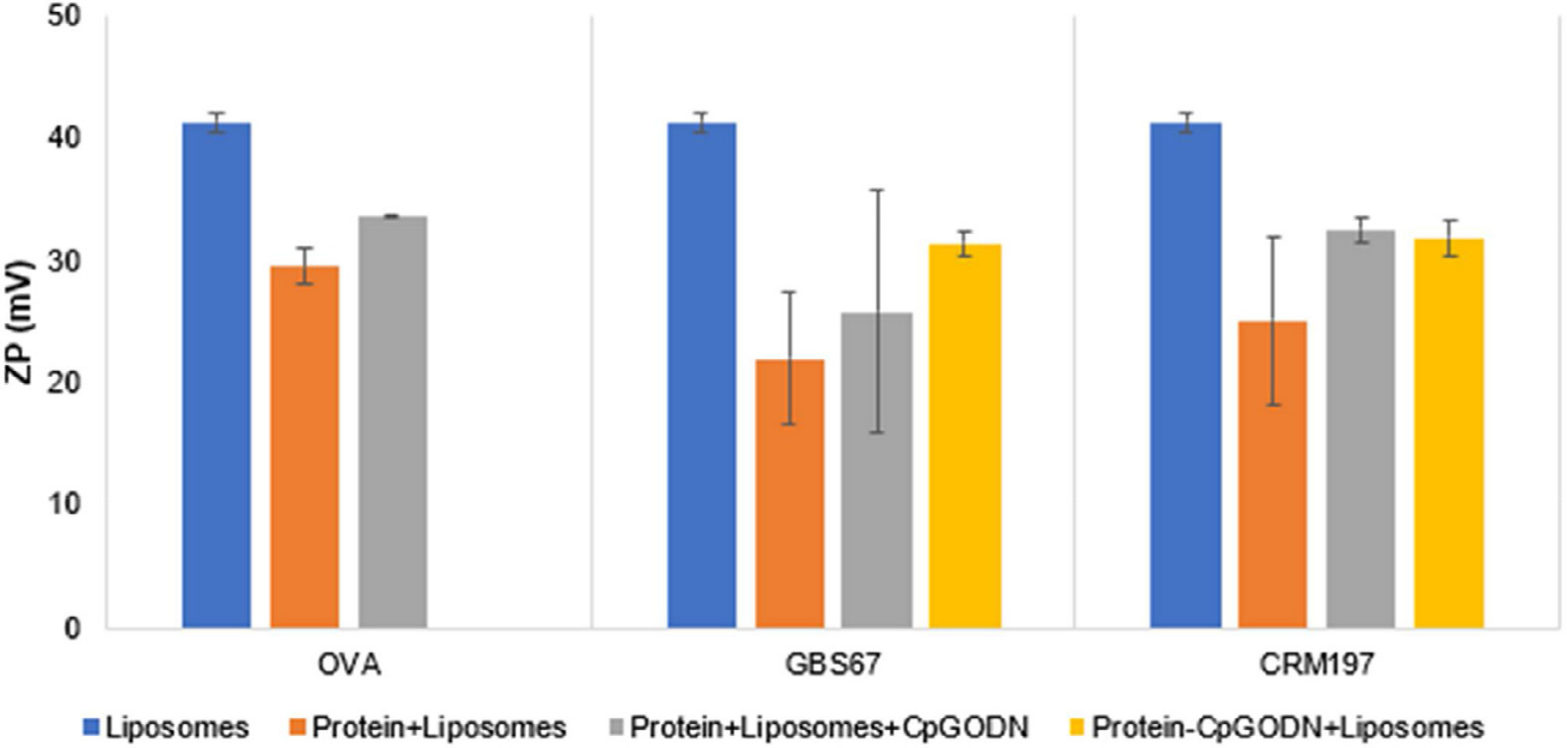
The effect of CpG ODN loading on zeta potential of liposomes. DSPC:Cholesterol:DDA (10:40:50% molar ratio) liposome were manufactured using microfluidics at 1:1 FRR, 12 mL/min TFR and purified using dialysis. Liposomes were mixed with free protein, protein+CpG ODN mixture or protein-CpG ODN conjugate and purified by dialysis. The final liposome (5 mg/mL), protein (0.25 mg/mL) and CpG ODN (0.038 mg/mL) concentrations in all the samples were the same. Liposomes were characterised in terms of zeta potential by DLS. Results represent mean ± SD, n = 3 independent batches.

Adsorption of the negatively charged protein and CpG ODN onto the cationic liposomes surface resulted in the increase of liposomes size and reduction of their surface charge as expected (Fig. 4). The highest increase in size was observed for the protein+liposomes+CpG ODN for all the proteins tested. Interestingly, no significant size increase was obtained when protein conjugate was mixed with DSPC: Cholesterol: DDA liposomes with the size remaining at 136 nm (Fig. 4). PDI values were lower than 0.3 across the formulation range tested which in conjunction with size distribution indicate uniform particles (Fig. 5). The lowest zeta potential measurements were observed when liposomes were mixed with protein alone for all the proteins (Fig. 6). On the other hand, when liposomes mixed with protein conjugates a 10 mV reduction was noticed from 41 mV to 31 mV for free liposomes and protein conjugates, respectively. Regarding the protein and CpG ODN loading on proteins, more than 90% protein and CpG ODN loading was achieved for all the formulations tested (Table 3).

**Table 3:** Protein and CpG ODN loading on liposomes. DSPC:Cholesterol:DDA (10:40:50% molar ratio) liposome were manufactured using microfluidics at 1:1 FRR, 12 mL/min TFR and purified using dialysis. Liposomes were mixed free with protein, protein+CpG ODN mixture or protein-CpG ODN conjugate and purified by dialysis. The final (5 mg/mL), protein (0.25 mg/mL) and CpG ODN (0.038 mg/mL) concentrations in all the samples were the same. Protein and CpG ODN quantification was carried out by BCA and UV, respectively. Results represent mean ± SD, n = 3 independent batches.

Protein	Formulation	Protein loading (%)	CpG ODN loading (%)
OVA	Protein+Liposomes	91 ± 3	–
	Protein+Liposomes+CpG ODN	93 ± 8	93 ± 9
	Protein-CpG ODN+Liposomes	–	–
GBS67	Protein+Liposomes	96 ± 3	–
	Protein+Liposomes+CpG ODN	95 ± 1	96 ± 5
	Protein-CpG ODN+Liposomes	95 ± 3	96 ± 1
CRM197	Protein+Liposomes	90 ± 3	–
	Protein+Liposomes+CpG ODN	92 ± 4	93 ± 4
	Protein-CpG ODN+Liposomes	96 ± 1	98 ± 1

## Conclusions

The potency of CpG ODN TLR9 agonist has been demonstrated by many researchers with some of its formulations being tested in clinical trials. CpG ODN has been used for stimulation of immune responses physically mixed with antigens and other adjuvants or encapsulated into nanoparticles for delivery to lymph nodes in an effort to protect it from degradation. Despite the research has been done so far, no research has been focused on the use of protein conjugates in conjunction with liposomes attributes. Conjugation efficiency has been proven extensively, especially when it is compared with simple co-administration of antigens and adjuvants [19]. It is supported that conjugation can ensure co-delivery of protein and adjuvant to the same cell [8]. Similarly, cationic liposomes use as adjuvants/delivery systems have attracted interest the last years due to their ability to absorb negatively charged molecules and their strong immunological properties [40,41]. Building on these evidences, this work aimed at designing a novel delivery system composed by the CpG ODN-protein complex and cationic liposomes, in an effort to maximise the vaccination potency.

Three different model proteins NadA, CRM197 and GBS67, have been successfully conjugated on CpG ODN motifs using maleimide-thiol chemistry as has been previously used for the preparation of other protein-CpG ODN conjugates [2,36]. Based on the isoelectric point of the proteins and their negative charge, cationic liposomes with DSPC: Cholesterol: DDA 10:40:50 composition and average size of 140 nm, demonstrated the capability to adsorb on their surface the negatively charged adjuvant-protein conjugate molecules to a very high degree. The conjugation approach as also the liposome contribution described in this study, can be particularly helpful for enhancement of immunity using low doses of antigen and increasing the speed of immunisations required to achieve effectiveness [42].

## CRediT authorship contribution statement

**Despo Chatzikleanthous:** Conceptualization, Visualization, Data curation, Formal analysis, Writing - original draft, Writing - review & editing. **Robert Cunliffe:** Conceptualization, Visualization, Data curation, Formal analysis, Writing - review & editing. **Filippo Carboni:** Conceptualization, Visualization, Data curation, Writing - review & editing. **Maria Rosaria Romano:** Conceptualization, Visualization, Writing - review & editing. **Derek T. O'Hagan:** Conceptualization, Visualization, Formal analysis, Writing - review & editing. **Craig W. Roberts:** Conceptualization, Visualization, Formal analysis, Writing - review & editing. **Yvonne Perrie:** Conceptualization, Visualization, Formal analysis, Writing - original draft, Writing - review & editing. **Roberto Adamo:** Conceptualization, Visualization, Formal analysis, Writing - original draft, Writing - review & editing.

## Declaration of Competing Interest

The authors declare the following financial interests/personal relationships which may be considered as potential competing interests: *FC, MRR, DO and RA are employees of the GSK group of companies. RA and DO are owners of GSK stocks. Other authors declare no conflict of interest.*

## Appendix Supplementary materials

Image, application 1
