# Supplementary material for: Synthesis of protein conjugates adsorbed on cationic liposomes surface
Source: MethodsX. 2020 May 28;7:100942. doi: 10.1016/j.mex.2020.100942 (PMC7289768; doi:10.1016/j.mex.2020.100942)
Supplement: Supplementary file 1 [file mmc1.docx]

**Supplementary material *and/or* Additional information**

***Synthesis of protein conjugates adsorbed on cationic liposomes surface***

*Despo Chatzikleanthous^1^, Robert Cunliffe^1^, Filippo Carboni^2^, Maria Rosaria Romano^2^, Derek T.O’Hagan^3^, Craig W. Roberts^1^, Yvonne Perrie^1^, Roberto Adamo^2*^.*

*^1^Strathclyde Institute of Pharmacy and Biomedical Sciences, University of Strathclyde, 161 Cathedral St, G4 0RE, Glasgow, UK*

*^2^GSK, Via Fiorentina 1, 53100, Siena, Italy*

*^3^GSK, 14200 Shady Grove Rd, Rockville, MD*

**Email:* [*roberto.x.adamo@gsk.com*](mailto:roberto.x.adamo@gsk.com)

MALDI-TOF of modified proteins pg. S2

^1^H NMR of modified CpG (D_2_O, 400 MHz) pg. S3

**MALDI-TOF of modified proteins**


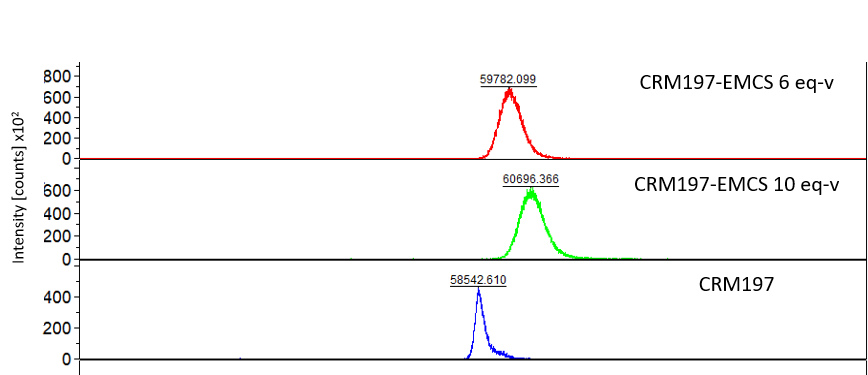


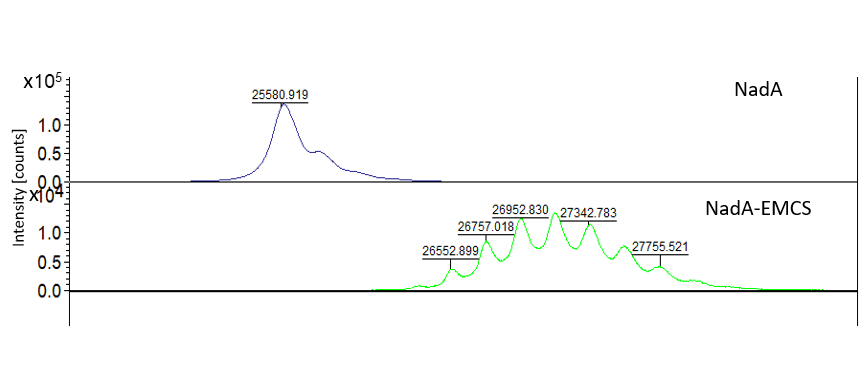


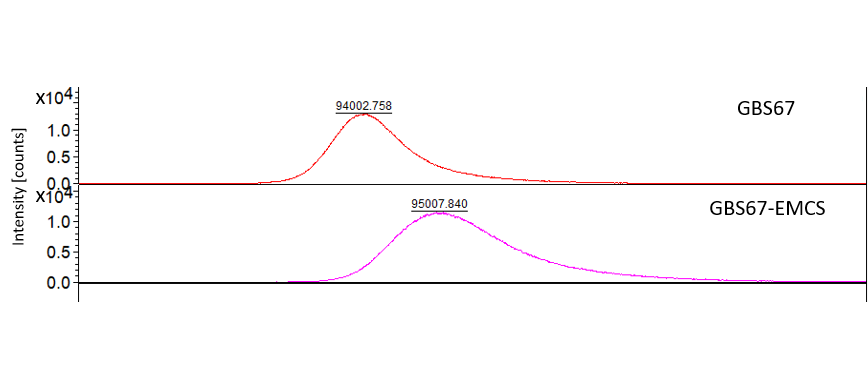


*Fig.S1 The linker/protein molar ratio was determined by MALDI-TOF mass spectrometry analysis run in an UltraFlex III MALDI-TOF/TOF instrument (Bruker Daltonics, Bremen, Germany) in linear mode and with positive ion detection.*

**^1^H NMR of modified CpG (D_2_O, 400 MHz)**


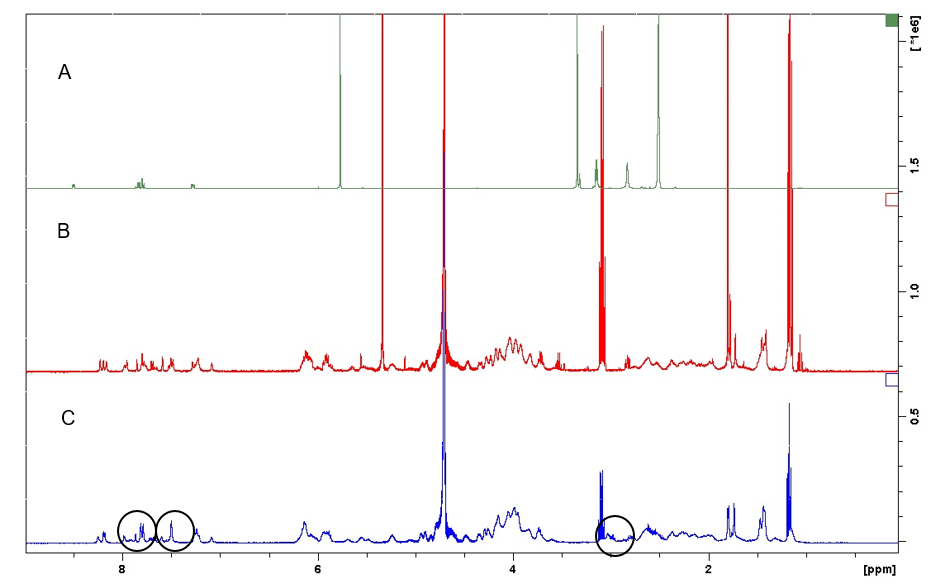


*Fig. S2.* *^1^H NMR analysis for the modification of CpG ODN with SPDP (A) SPDP (B) CpG ODN (C) CpG ODN-SPDP.*
